# Medical dispatchers’ experience with live video during emergency calls: a national questionnaire study

**DOI:** 10.1186/s12913-024-11939-4

**Published:** 2024-11-20

**Authors:** Natascha H Bohnstedt-Pedersen, Gitte Linderoth, Barbara Helios, Helle C Christensen, Britta K Thomsen, Lisbeth Bekker, Jannie K B Gram, Ulla Vaeggemose, Tine B Gehrt

**Affiliations:** 1 Department of Research & Development, Prehospital Emergency Medical Services, Central Denmark Region, Aarhus, Denmark; 2 Copenhagen Emergency Medical Services, University of Copenhagen, Copenhagen, Denmark; 3 Department of Anesthesia and Intensive Care, Copenhagen University Hospital - Bispebjerg and Frederiksberg, Copenhagen, Denmark; 4 Zealand Emergency Medical Services, Institute of Clinical Medicine, University of Copenhagen, Naestved, Denmark; 5 Zealand Emergency Medical Services, Naestved, Denmark; 6 Emergency Medical Services, North Denmark Region, Aalborg, Denmark; 7 Department of Politics and Society, Aalborg University, Aalborg, Denmark; 8 Department of Clinical Medicine, Aarhus University, Aarhus, Denmark; 9 Department of Research and Development, Central Denmark Region, Prehospital Emergency Medical Services, Brendstrupgårdsvej 7, 2. th, Aarhus N, 8200 Denmark

**Keywords:** Emergency Medical Service, EMS dispatcher, Emergency Medical Dispatch, Telephone triage, Telemedicine, Telehealth, Video

## Abstract

**Background:**

Telehealth has become increasingly essential in healthcare provision, also in the Prehospital Emergency Medical Services (EMS), where live video is implemented as a supplemental tool to assess and triage medical emergency calls. So far, using video for emergency calls seems beneficial for patient assessment and dispatcher-assisted first aid. However, the EMS dispatchers’ experiences with and perceptions of using video during emergency calls are largely unexplored.

**Methods:**

In 2023, a nationwide survey study was conducted in Denmark, which is covered by five Emergency Medical Dispatch Centers. All Danish EMS dispatchers were invited to participate in the study. The survey explored the dispatchers’ experience with using video during emergency calls, the perception of their own video use, and the process of implementing video as a new tool in their working procedure. Main questions were answered on a scale from 1 to 7, where higher scores indicate more agreement.

**Results:**

Of the 183 EMS dispatchers employed during the study period, 78% completed the survey. They found video easy to use (median = 7) and found video supportive in guidance and dispatch when the patient’s problem was unclear (median = 7), but did not find video suitable for all emergency calls and expressed that complications with the technology was a barrier for using video. The EMS dispatchers were least likely to agree that they choose not to use video due to the risk of being emotionally affected by what they might see (median = 1). When dividing the sample based on EMS dispatcher’s gender, age, seniority, and educational background, generally few differences between groups were found.

**Conclusions:**

Live video during emergency calls is generally experienced as a useful supplemental tool by EMS dispatchers in Denmark, and the greatest self-perceived barriers for using video were not finding video suitable for all situations and the technology.

**Supplementary Information:**

The online version contains supplementary material available at 10.1186/s12913-024-11939-4.

## Background

Telehealth has become increasingly used in healthcare provision worldwide to solve present issues and improve care [1–5]. Telemedicine and live video are telehealth solutions that have been applied in many clinical settings in Northern Europe and the USA, e.g. primary care and outpatient clinics to provide remote consultations and in out-of-hours medical helplines to assess and triage patients [6–13]. Live video in various configurations and set-ups has also become increasingly applied in the Emergency Medical Services (EMS) to support the prehospital healthcare providers in the field with remote assistance [14–17]. Furthermore, many EMS providers have integrated live video transmission in the Emergency Medical Dispatch Centers (EMDC), that handle health-related emergency calls [18–23]. Video is in the EMDCs implemented as a supplemental tool (in addition to telephone contact) for the EMS dispatchers to assess and triage medical emergency calls [19–25].

Existing knowledge on the use of video during emergency calls mainly comes from clinical studies and simulation studies showing that video is a valuable tool for EMS dispatchers to assess patients, facilitate and correct dispatcher assisted cardiopulmonary resuscitation, and dispatch of emergency responses [19–34]. Furthermore, one study found that EMS dispatchers classified fewer emergency calls as being related to an ‘unclear problem’ when using video compared to audio-only emergency calls [23]. Taken together, these findings suggest that video during emergency calls improves the healthcare provided by the EMS.

Interview studies have investigated EMS dispatcher’s experiences with video and explored their perception of what happened on scene and the value of visual information in real out-of-hospital cardiac arrest [35, 36]. These studies showed that visual information during emergency calls contributed to improve the EMS dispatchers understanding of the situation, being more comfortable with their decision-making, enhance communication and provide better guidance to the bystanders [35, 36]. Furthermore, a questionnaire study explored bystanders’ perception of video-use during emergency calls and found that this was generally experienced as beneficial and that the majority of the bystanders were very satisfied with the help they received from the EMS dispatchers when video was employed [23]. These qualitative studies add to the quantitative studies reviewed above, by demonstrating that bystanders were also positive about the use of video during emergency calls and that video was also perceived by the dispatchers to improve the healthcare they provide. The perception of the EMS dispatchers on the value of video during emergency calls is essential, as they are the primary users and crucial stakeholders of the usage of video. However, there are only two previous studies focusing on the EMS dispatchers’ perception of using video during emergency calls and the specific case of using video for emergency calls involving out-of-hospital cardiac arrest. Furthermore, these studies only included a small number of EMS dispatchers.

To begin to fill the knowledge gap in the existing literature, the present study presents the results of a nationwide survey sent to all Danish EMS dispatchers examining the dispatchers’ experiences with and self-perceived use of video during emergency calls.

## Methods

### Setting

Denmark is a Scandinavian country covering an area of 43.000 square kilometers with a population of 5.9 million inhabitants [37]. Access to the Danish healthcare system is mainly free of charge for all residents [38–40]. The country is divided into five regions that are responsible for running a regional Prehospital EMS and a regional EMDC, which is the point of entry for the EMS and the citizens’ access to immediate medical help in emergency situations through the national emergency phone number “1-1-2” [41]. In 2023, a total of 442.856 emergency calls were handled by the five Danish EMDCs.

In the case of a health-related emergency, the caller is seamlessly transferred to the EMDC through a central call center managed by the police [41]. The EMS dispatchers assess, process, and respond to the call by initiating an EMS response and providing medical guidance over the phone [41]. The EMS dispatchers are registered nurses or paramedics, and their decision-making process is guided by a nationwide criteria-based emergency medical dispatch index [42].

A technical solution for video transmission during emergency calls was implemented from 2018 to 2023 in all five EMDCs in Denmark. Three different providers are responsible for the technical solution. The video solution is integrated into the dispatch system in one EMDC, whereas it is provided on an external platform in the other four EMDCs. All three video solutions work by the EMS dispatcher sending a text message to the caller asking the caller for consent to share video from the camera on their smartphone. After confirmation, a secure one-way video transmission from the scene to the EMS dispatcher is feasible. This video solution is ideal in Denmark where video-capable smartphones are widespread and more than 90% of adults in Denmark own such a device [43].

## Procedure and participants

We used a cross-sectional survey design to investigate the EMS dispatchers self-perceived use of video during emergency calls. Data was collected from 1st June 2023 to 31st August 2023 using the Electronic Data Capture system REDCap [44, 45]. An invitation e-mail was sent to all 183 EMS dispatchers who were employed the 1st June 2023 in one of the five EMDCs in Denmark. This e-mail contained information about the aim of the study and a direct link to the electronic REDCap questionnaire. A reminder about the study was sent to all EMS dispatchers two weeks later and a second reminder two months after the initial invitation. The EMS dispatchers were additionally verbally informed about the study in the EMDC in which they were employed. Participation in the study was voluntary. In the questionnaire, participants were first presented with a data protection statement and information about the study’s purpose, followed by an informed consent form. Participants then answered the questionnaire described below, and finally were presented with a debriefing statement thanking them for their participation and providing contact information to the study group.

## Questionnaire

The questionnaire was purposely developed for this study and is provided in the supplementary materials. Items of the questionnaire were based on insights from a literature search and data from interviews with EMS dispatchers in the Central Denmark Region, Denmark. The drafting process was based on the following steps: (1) Literature search for research on the use/implementation of video/technology between a healthcare professional and a layperson/patient/caller and which barriers they experienced when using video as a supplementary healthcare tool, as well as interview data concerning EMS dispatcher’s attitudes towards using video during emergency calls; (2) The first version of the survey was developed and the draft was sent to the research group, discussed at a joint meeting, and subsequently revised; (3) EMS dispatchers from three EMDCs pilot tested and validated the revised questionnaire; (4) Final revision of the questionnaire based on comments from the research group and insights from the pilot testing; (5) The final questionnaire was approved by the research group.

The final questionnaire contained 31 items that were divided into six domains: [1] basic demographics (age, gender, educational background, seniority, region of employment) [2], the EMS dispatchers’ opportunity to use video during emergency calls and estimation of own video use (2 items) [3], the implementation of the video solution and working procedure (7 items) [4], their experiences with video during emergency calls (10 items) [5], their use of video during emergency calls (6 items), and [6] the biggest self-perceived barrier for using video during emergency calls (1 item; participants had to select one of 15 response options). At the end of the questionnaire, the EMS dispatchers were able to add comments in an open-ended text box. The respondents answered the questions concerning the implementation of video, their experiences with video and use of video (domain 3, 4 and 5) using a 7-point Likert scale (1 = strongly disagree, 4 = neutral, 7 = strongly agree). Participants were required to fill out all items in the questionnaire in order to prevent missing data.

### Data analysis

Data were analyzed using Stata version 18.0 MP-parallel edition [46]. All incomplete questionnaires were removed from the final dataset before analysis to prevent double registrations or other influence of errors from the incomplete questionnaires on the data. Differences between groups (based on gender, age, seniority, and professional training) on answers to individual items were tested with independent group t-tests. All *p*-values are two-tailed and considered statistically significant if < 0.05. Although t-test are valid on non-normally distributed data if the sample size is large enough [47, 48], the same group differences were examined with a non-parametric Mann-Whitney test and reported in the supplemental online material, as not all data was normally distributed. For tests of group differences based on professional training, personnel who received their educational training in an ambulance (paramedic, ambulance technician) are categorized as ambulance personnel and personnel who received their educational training in the hospital (nurses, midwifes) are categorized as intra-hospital personnel. Participants’ comments from the open-ended text box were subject to thematic analysis, placing the comments into categories with certain themes in common.

## Results

### Participants

 In the study period, 183 EMS dispatchers were employed in Denmark, 174 opened the link to the questionnaire, and 144 completed the entire questionnaire. Thus, the response rate was 78.7%. The majority of the EMS dispatchers were female, nurses, and had more than 5 years of seniority. Further participant characteristics including self-evaluated estimation of video use are shown in Table 1.

**Table 1:**
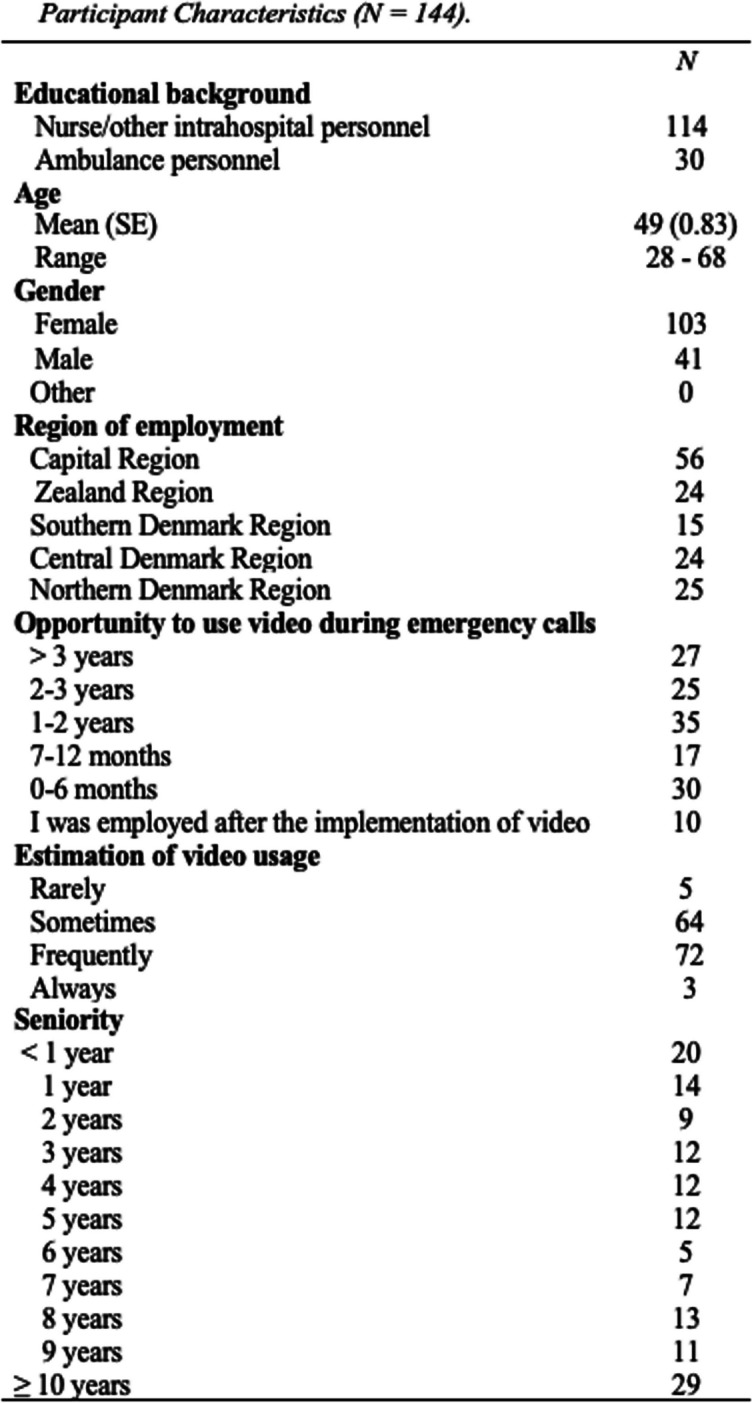
....

## Questionnaire

 The wording and descriptive statistics for all items concerning experiences with and perceptions of the use of video are presented in Table 2.

**Table 2:**
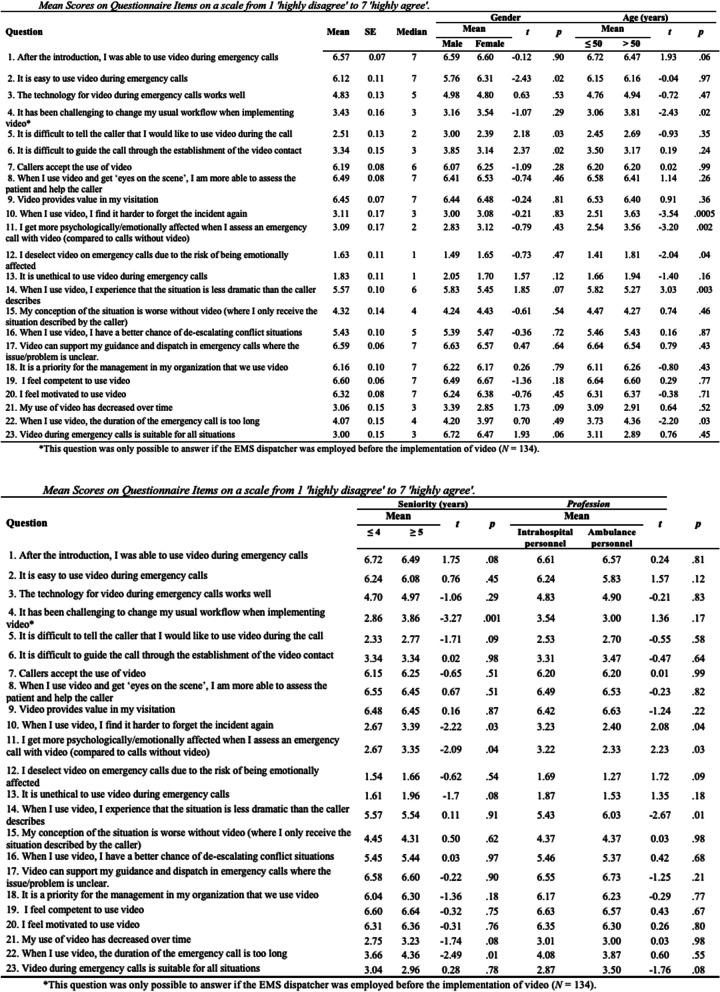
.....

The EMS dispatchers were generally positive about using video during emergency calls. They found video easy to use (mean = 6.12; median = 7) and indicated that video can support their guidance and dispatch in emergency calls when the patient’s problem is unclear (mean = 6.59; median = 7). The EMS dispatchers were least likely to agree that they chose not to use video due to the risk of being emotionally affected by what they might see (mean = 1.63; median = 1) and that it is unethical to use video for emergency calls (mean = 1.83; median = 1).

Questions regarding the implementation of video concerned the introduction to use video during emergency calls, the technology, workflow and communication with the caller about using video. The EMS dispatches agreed that the introduction enabled them to use video (mean = 6.57; median = 7) and that video is easy to use (mean = 6.12; median = 7). They did not find it difficult to communicate with the caller about using video during the emergency call (mean = 2.51; median = 2), neither was it difficult to guide the caller through the establishment of the video connection (mean = 3.34; median = 3). The EMS dispatchers were not completely content with the video solution available to them (mean = 4.83; Median = 5), but did not have major difficulties changing their usual workflow when using video during emergency calls (mean = 3.43; median = 3).

The EMS dispatchers find themselves abler to assess the patient and help the caller when using video (mean = 6.49; median = 7) and agree that video adds value in their triage of emergency calls (mean = 6.45; median = 7). The results show that EMS dispatchers generally don’t find that they become more psychologically affected when assessing emergency calls using video (mean = 3.09; median = 2) and they do not find it harder to forget an incident again when video was used during the emergency call (mean = 3.11; median = 3). Also, EMS dispatchers experience situations as less dramatic when they use video (mean = 5.57; median = 6) and they find their conception of the situation worse when they do not use video and only can assess the situation as described by the caller (mean = 4.32; median = 4). Another positive finding is that EMS dispatchers see video as a helpful tool for de-escalating conflicts with the caller (mean = 5.43; median = 6).

Furthermore, the EMS dispatchers feel both competent (mean = 6.60; median = 7) and motivated to use video (mean = 6.32; median = 7), as well as that the management in their organization prioritize their use of video during emergency calls (mean = 6.16, median = 7). They do not feel that their use of video has decreased over time (mean = 3.06; median = 3) and have a neutral attitude towards whether the duration of emergency calls was too long when they use video (mean = 4.07; median = 4).

## Greatest barrier for the use of video

 The EMS dispatchers were asked to select the greatest barrier for their use of video during emergency calls from a list of 15 possible options. A total of 51 (35%) EMS dispatchers indicated that the greatest barrier for using video is that video is not suitable for all situations, 31 (22%) answered that difficulties with the technology is the ultimate barrier for using video, and 16 (11%) EMS dispatchers answered that they do not need video to assess and dispatch emergency calls. See Table 3 for an overview of all possible response options for this question and the results for how often each potential barrier was endorsed.

**Table 3:**
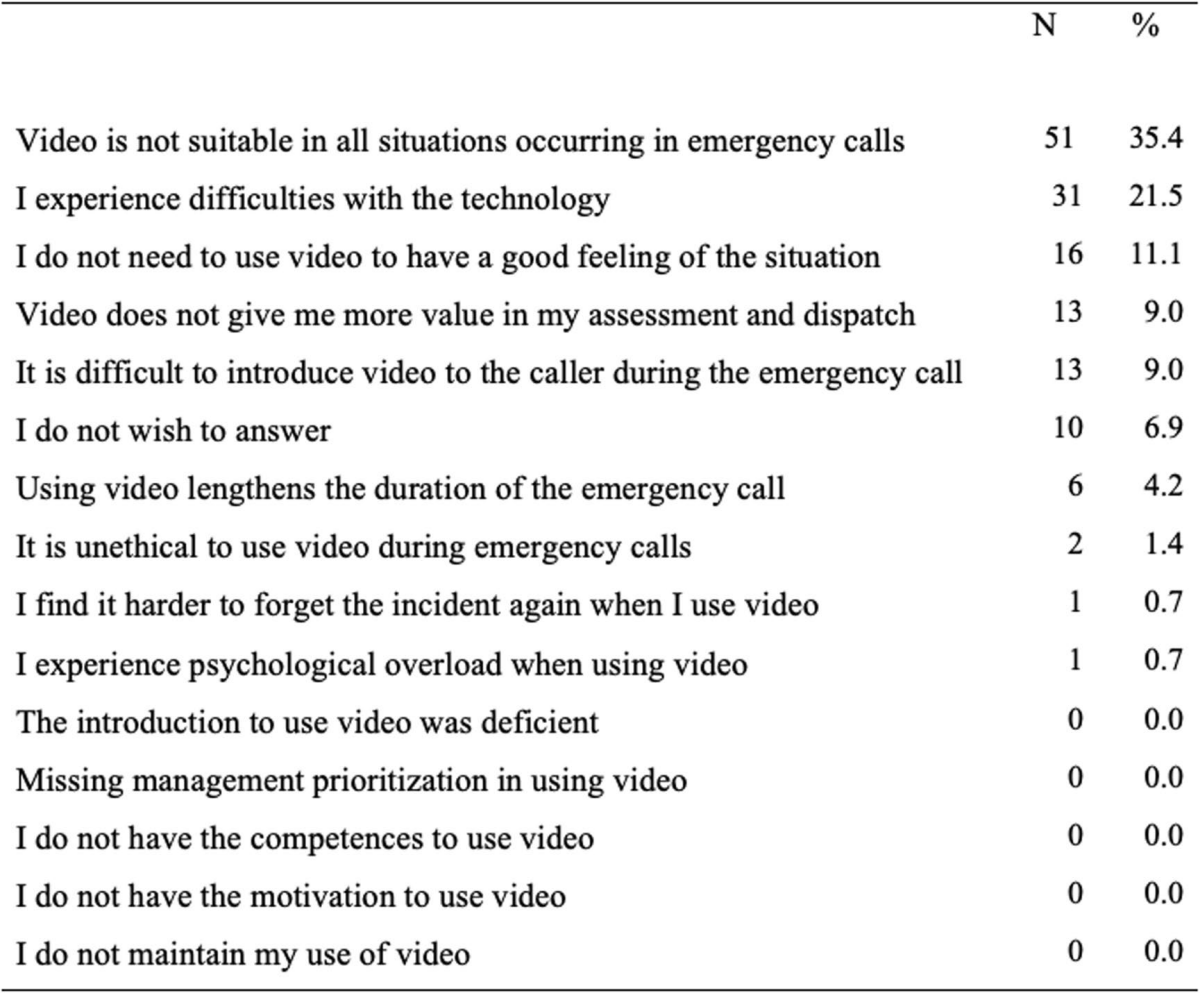
...

### Thematic analysis of the open-ended question

**Fig. 1:**
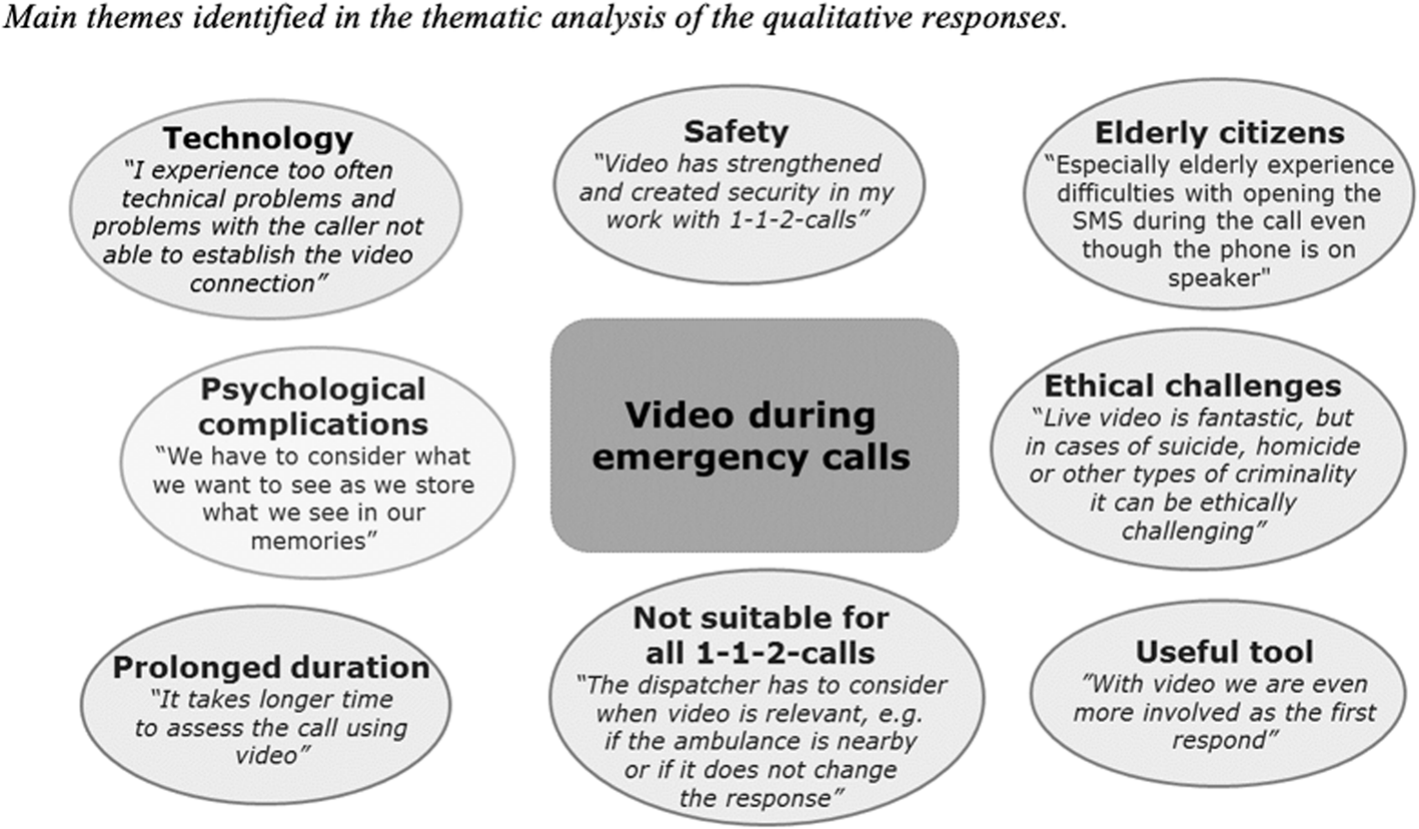
...

Of the 144 EMS dispatchers completing the questionnaire, 52 (36%) added a comment in the open-ended text box, revealing different perspectives of and insights into using video during emergency calls. Coding and thematic analysis was performed to identify central themes among the responses. The thematic analysis identified eight main themes: [1] Technology, which concerns the video-setup and complications with the connection to the caller’s smartphone camera (identified in 27 responses) [2], video creates a sense of safety in the assessment and dispatch of emergency calls (identified in 3 responses) [3], video as a useful tool enabling better insight into the situation and guidance, especially when the caller is not able to describe the patient’s condition and the situation (identified in 12 responses) [4], video is not suitable for all emergency calls (identified in 6 responses) [5], video results in prolonged duration of emergency calls (identified in 2 responses) [6], video is not suitable for elderly people and when there are language barriers (identified in 5 responses) [7], how using video during emergency calls psychologically affects EMS dispatchers (identified in 2 responses) and [8] ethical challenges, specifically concerning situations where the use of video is experienced as ethically complicated (identified in 2 responses). See Fig. 1 for an overview of the identified themes and a quote illustrating each theme.

### Differences between groups

To test if participants’ gender, age, seniority or professional background influenced their responses, independent group t-tests were performed (see Table 2).

The same analyses were performed with a non-parametric Mann-Whitney test, which yielded similar results as the t-tests with few exceptions (see Supplementary Table 1). Overall, there were generally few differences between the groups in their responses to the items of the questionnaire.

When dividing the EMS dispatchers based on self-selected gender (an ‘other’ option was available, but not chosen by any participants), women found it easier to use video compared to men (mean: 6.31 versus 5.76, *p* = .02). Similarly, men found it more difficult to introduce video to the caller (mean: 3.00 versus 2.39, *p* = .03) and to guide the caller through the video establishment (mean 3.85 versus 3.14, *p* = .02) compared to women.

Regarding seniority, EMS dispatchers employed for more than five years in an EMDC self-reported that it had been challenging to change their working procedure when video was implemented (3.86 versus 2.86, *p* = .01) and that the duration of emergency calls using video is too long (mean: 4.36 versus 3.66, *p* = .01) compared to EMS dispatchers employed for less than five years.

EMS dispatchers above the age of 50 also felt that the duration of emergency calls using video was too long (mean: 4.36 versus 3.73, *p* = .03), that it had been challenging to change their working procedure (mean: 3.81 versus 3.06, *p* = .02), and that the emergency situation is less dramatic than the callers’ initial description when using video (mean: 5.58 versus 5.27, *p* = .03) compared to EMS dispatchers less than 50 years of age.

The only statistically significant differences between the groups with different educational background was that ambulance personnel found the emergency situation less dramatic than the callers’ initial description when using video (mean 6.03 versus 5.43, *p* = .01) compared to intra-hospital personnel.

### Regional differences

When dividing the EMS dispatchers by their EMDC of employment and comparing the responses to the items of the questionnaire, the means for each region were generally very similar. There was only one item (the question concerning the duration of emergency calls when using video), where the mean response for the regions were on different sides of the neutral (response option 4) on the Likert Scale. That is, EMS dispatchers from two EMDC’s agreed that the duration of emergency calls is too long when using video (means: 4.21 and 4.92), whereas EMS dispatchers from three EMDC’s disagreed with this statement (means: 3.38, 3.60 and 3.96). See Supplementary Table 2 for the means for each Danish region separately.

## Discussion

We conducted the first national survey on EMS dispatchers’ experiences with and perceptions of using video during emergency calls in Denmark. A high response rate was achieved (78.7% of all Danish EMS dispatchers). We found that Danish EMS dispatchers in general were positive about using video during emergency calls and found video easy to use and feel that it supports their guidance and dispatch in situations where the callers’ problem is unclear. The EMS dispatchers highly disagree that video is unethical to use and that they actively deselect video in emergency calls due to the risk of being emotionally affected by what they might see. There were in general only few statistically significant differences between groups, when we stratified the EMS dispatchers by gender, age, seniority and professional background. Previous studies have found that video is easy to use during emergency calls, that callers accept video streaming, and that EMS dispatchers find video as helpful in order to visualize the patient or the scene to get a better impression of the situation [22, 23, 36]. The present study adds to the existing literature on the use of video during emergency calls by demonstrating factors affecting the EMS dispatchers use of video.

The ratings of the questionnaire items were quite similar across the five EMDC’s in Denmark and their responses were only on different sides of the neutral option in the question concerning the duration of emergency calls when using video. Another interesting finding concerned the question related to the technology used to provide video streaming. The EMS dispatchers in the Capital Region of Denmark rated that the technology for using video during emergency calls works better compared to the other four EMDCs (see Supplementary Table 2). In the Capitol Region of Denmark, the video solution was integrated into the dispatch system whereas in the other EMDCs the system for handling video calls was provided on an external platform. This integration into the dispatch system might have a positive effect on video use and prevent some of the complications the EMS dispatchers’ experience with using video. That issues with the technology might be a key component for using video (or not) during emergency calls is also underscored by the fact that the second most often endorsed main barrier for using video was the technology, and that 27 of the 52 comments in the free-text box regarded problems with the technology, of which one example was the following quote *“Easy and fast establishment of the video connection and optimal video quality is very important”*. This barrier was also seen in other studies showing that technical training and support are important factors regarding an implementation of telemedicine in the acute care setting [5, 49]. These findings highlight the need to implement video solutions that are easy to use, not only for the callers, but also for the EMS dispatchers.

Another interesting finding was that EMS dispatchers employed for more than five years found it somewhat harder to forget incidents from emergency calls when using video compared to EMS dispatchers employed for less than five years. This study was the first to investigate seniority as a factor regarding the use of video during emergency calls but this finding was contrary to that one might expect that higher seniority means more exposure to extremely negative emergency calls, which could make the EMS dispatchers more robust in handling all kinds of emergency calls, regardless of whether they used video or not. However, EMS dispatchers could be at risk of developing secondary traumatization due to the indirect exposure to traumatic events during emergency calls [50–53]. When using video, there could be a risk of this indirect exposure becoming more intense, which could affect EMS dispatchers with more years of service more due to the longer duration of exposure. Another explanation could be that high-seniority EMS dispatchers might have distanced themselves more from the clinical reality of visually assessing patients after working with telephone-only emergency calls for many years. EMS dispatchers with employment for less than five years, however, had less experience with telephone-only emergency calls or were employed after the implementation of video streaming. This could result in a less integrated telephone-only dispatch working procedure and make them more prepared to also handle the visual information during the emergency call that video provides.

Furthermore, all EMS dispatchers agreed that using video during emergency calls aided better communication with the caller, including fewer conflicts between the EMS dispatcher and the caller during the emergency call. This was also reflected in the tests for group differences, with all means being close to the positive maximum and no group differences being statistically significant for this item. In combination with the finding that EMS dispatchers agreed that video supported their guidance and dispatch in emergency calls where the problem was unclear, these findings indicated that video contributed to solve general miscommunication between the EMS dispatcher and the caller [23]. Individuals who call the EMDC range in emotional stages from being extremely distressed to calm and focused. The callers’ emotional stage often affects the communication and the emergency call handling [54]. The quality of the information given to the EMS dispatcher was likewise likely affected by the callers’ attitude, use of words and language, and willingness to assess the patient [54]. These parameters were important for the EMS dispatchers for correct handling of the emergency call and dispatch of emergency response. The use of video made the EMS dispatcher less dependent to the callers’ verbal information because of the ability to have ‘eyes on the scene’ and assess the patient using two senses; the sense of hearing and sight.

Lastly, individuals making emergency calls have expressed that it was important for them to feel that they were taken seriously by the EMS dispatcher [55]. Emergency callers expected to receive help when calling the EMDC and most callers had the impression that this equaled an ambulance arriving or being admitted to the hospital, although an ambulance was not needed in many of the emergency calls handled by the EMDCs [55]. When EMS dispatchers used video, they had the ability to meet the caller with clearer communication when seeing the patient and to assure the caller that an ambulance was not needed or assure the caller they were providing the right first aid which could prevent the caller from arguing for more help or feeling dissatisfied and rejected by not getting an ambulance.

The present study had several strengths. First, this study was the first to investigate the EMS dispatchers use of video though a cross-sectional survey design with almost 80% of the entire population of EMS dispatchers in Denmark completing the questionnaire, which ensures the generalizability of the results. However, we had no information about the little more than 20% that did not complete the questionnaire (e.g., age, professional background), hence we must assume there was selection bias. Second, the questionnaire was sent out to all EMS dispatchers by their employer, hence they could have felt pressure to participate. However, the questionnaire was answered anonymously and the raw data was only available for two researchers from Central Denmark Region, who were not employed in an EMDC. The data was never shared with the employers or the management, which the participants were informed about. Thus, the bias brought about by this is expected to be minimal. Third, the only other study investigating the use of video during emergency calls was a qualitative study with a sample size of 25 participants, hence the present study is more comprehensive [35]. Fourth, the questionnaire was developed after extensive work including literature search, interviews, several pilot tests, and revisions by the research group. This procedure was made in the absence of other validated questionnaires regarding the use of video during emergency calls. A questionnaire was chosen as it allowed us to assess many aspects of the use of video through the different items and to get ratings (on a Likert scale) that could be compared based on e.g. EMS dispatchers’ gender or place of employment. However, a qualitative study would have allowed the EMS dispatchers to elaborate on their perceptions of and experiences with using video for emergency calls. In this study, we did provide participants the option to write comments in a free text box, which more than a third of the EMS dispatchers did. Fifth, the EMS dispatchers agreed with the statement ‘video is not suitable in all situations occurring in emergency calls’, which indicates that the use of video depends on the nature of the situation that caused the emergency call. This selective use of video strengthens their perception of where video is most valuable, which is important for the acceptance of video but may also limit the full potential of video. Future studies should further examine the potential selective use of video in emergency calls.

## Conclusion

EMS dispatchers in Denmark were generally positive about using video for emergency calls, but they were bothered by difficulties with the technology and did not find video suitable for all emergency calls. The EMS dispatchers found video valuable in situations where the problem was unclear and did not perceive video as a risk factor for being emotionally affected by an emergency call. Neither did they find it unethical to use video during emergency calls. Differences between groups of EMS dispatchers divided by gender, age, seniority, and professional background were few, and mostly concerned the change of working procedures, introduction of video and guidance of the caller, and the perception of the emergency call as less dramatic when getting ‘eyes on the scene’ through video.

## Supplementary Information

Supplementary material 1.

Supplementary material 2.

## Data Availability

The dataset supporting the conclusions of this article has not been made available on a permanent third-party archive given the nature of the data (i.e., participants’ life stories) and General Data Protection Regulations (GDPR). However, access to the data (in anonymized form) will be granted from the corresponding author upon request, but may require the completion of a formal data sharing agreement, in compliance with GDPR and Aarhus University and the Central Denmark Region rules. The employed materials are available through the Methods section.
