# Supplementary material for: Medical dispatchers’ experience with live video during emergency calls: a national questionnaire study
Source: BMC Health Serv Res. 2024 Nov 20;24:1442. doi: 10.1186/s12913-024-11939-4 (PMC11577824; doi:10.1186/s12913-024-11939-4)
Supplement: Supplementary file 1 — Supplementary material 1. [file 12913_2024_11939_MOESM1_ESM.docx]

**Supplementary Table 1**

Mann-Whitney test of group differences on questionnaire items rated on a scale from 1 'highly disagree' to 7 'highly agree'.

|  | **Gender** | | | | | | | **Age (years)** | | | | | | |  |
| --- | --- | --- | --- | --- | --- | --- | --- | --- | --- | --- | --- | --- | --- | --- | --- |
|  | **Rank sum** | | | | ***z*** | ***p*** | | **Rank sum** | | | | ***z*** | | ***p*** | |
|  | **Male** | | **Female** | |  |  |  | **≤ 50** | | | **> 50** |  |  |  |  |
| 1. After the introduction, I was able to use video during emergency calls | 2925 | 7515 | | -.27 | | | .79 | | 56814.5 | 4758.5 | | 1.98 | .048 | |  |
| 2. It is easy to use video during emergency calls | 2640.5 | 7799.5 | | -1.60 | | | .11 | | 5337 | 5103 | | .20 | .84 | |  |
| 3. The technology for video during emergency calls works well | 3093 | 7347 | | -.55 | | | .59 | | 5044.5 | 5395.5 | | -1.01 | .31 | |  |
| 4. It has been challenging to change my usual workflow when implementing video* | 2268 | 6777 | | -1.16 | | | .25 | | 3963.5 | 5081.5 | | -2.22 | .03 | |  |
| 5. It is difficult to tell the caller that I would like to use video during the call | 3600.5 | 6839.5 | | 2.88 | | | .004 | | 5086.5 | 5353.5 | | -.85 | .39 | |  |
| 6. It is difficult to guide the call through the establishment of the video contact | 3499.5 | 6940.5 | | 2.37 | | | .02 | | 5626 | 4814 | | 1.36 | .18 | |  |
| 7. Callers accept the use of video | 2789.5 | 7650.5 | | -.88 | | | .38 | | 5209.5 | 5230.5 | | -.36 | .72 | |  |
| 8. When I use video and get ‘eyes on the scene’, I am more able to assess the patient and help the caller | 2863.5 | 7576.5 | | -.59 | | | .55 | | 5444.5 | 4995.5 | | .75 | .45 | |  |
| 9. Video provides value in my visitation | 3010 | 7430 | | .20 | | | .85 | | 5511.5 | 4928.5 | | 1.02 | .31 | |  |
| 10. When I use video, I find it harder to forget the incident again | 2875 | 7565 | | -.44 | | | .66 | | 4351.5 | 6088.5 | | -3.86 | .001 | |  |
| 11. I get more psychologically/emotionally affected then I assess an emergency call with video (compared to calls without video) | 2794.5 | 7645.5 | | -.81 | | | .42 | | 4428 | 6012 | | -3.54 | .004 | |  |
| 12. I deselect video on emergency calls due to the risk of being emotionally affected | 2877 | 7563 | | -.53 | | | .60 | | 4975 | 5465 | | -1.57 | .12 | |  |
| 13. It is unethical to use video during emergency calls | 3131 | 7309 | | .80 | | | .43 | | 4925.5 | 5514.5 | | -1.66 | .10 | |  |
| 14. When I use video, I experience that the situation is less dramatic than the caller describes | 3356.5 | 7083.5 | | 1.79 | | | .07 | | 5850.5 | 4589.5 | | 2.34 | .02 | |  |
| 15. My conception of the situation is worse without video (where I only get the situation described by the caller) | 2921 | 7519 | | -.23 | | | .82 | | 5602.5 | 4837.5 | | 1.27 | .21 | |  |
| 16. When I use video, I have a better chance of de-escalating conflict situations | 2905 | 7535 | | -.31 | | | .76 | | 5328.5 | 5111.5 | | .15 | .88 | |  |
| 17. Video can support my guidance and dispatch in emergency calls where the issue/problem is unclear. | 3032.5 | 7407.5 | | .33 | | | .74 | | 5319.5 | 5120.5 | | .13 | .89 | |  |
| 18. It is a priority for the management in my organization that we use video | 2937.5 | 7502.5 | | -.17 | | | .86 | | 5069 | 5371 | | -.99 | .32 | |  |
| 19. I feel competent to use video | 2883 | 7557 | | -.51 | | | .61 | | 5425 | 5015 | | .68 | .50 | |  |
| 20. I feel motivated to use video | 2935.5 | 7504.5 | | -.19 | | | .85 | | 5309.5 | 5130.5 | | .08 | .94 | |  |
| 21. My use of video has decreased over time | 3366.5 | 7073.5 | | 1.78 | | | .07 | | 5483 | 4957 | | .78 | .44 | |  |
| 22. When I use video, the duration of the emergency call is too long | 3111 | 7329 | | .62 | | | .53 | | 4776.5 | 5663.5 | | -2.10 | .04 | |  |
| 23. Video during emergency calls is suitable for all situations | 3265.5 | 7174.5 | | 1.32 | | | .19 | | 5624 | 4816 | | 1.35 | .18 | |  |

*This question was only possible to answer if the EMS dispatcher was employed before the implementation of video (*N* = 134).

**Supplementary Table 1 – Continued**

|  | **Seniority (years)** | | | | | | | | **Profession** | | | | | | |  |
| --- | --- | --- | --- | --- | --- | --- | --- | --- | --- | --- | --- | --- | --- | --- | --- | --- |
|  | **Rank sum** | | | | ***z*** | ***p*** | | | **Rank Sum** | | | ***z*** | | ***p*** |  |  |
|  | **≤ 4** | | **≥ 5** | |  |  |  |  | **Nurse / intrahospital personnel** | | **Ambulance personnel** |  |  |  |  |  |
| 1. After the introduction, I was able to use video during emergency calls | 5258.5 | 5181.5 | | 2.05 | | | .04 | 8377.5 | | 2062.5 | | .71 | .48 | | | |
| 2. It is easy to use video during emergency calls | 4940 | 5500 | | .36 | | | .72 | 8538 | | 1902 | | 1.48 | .14 | | | |
| 3. The technology for video during emergency calls works well | 4592 | 5848 | | -1.09 | | | .28 | 8195 | | 2245 | | -.35 | .73 | | | |
| 4. It has been challenging to change my usual workflow when implementing video* | 6150 | 5895 | | -3.18 | | | .002 | 7552.5 | | 1492.5 | | 1.5 | .13 | | | |
| 5. It is difficult to tell the caller that I would like to use video during the call | 4483 | 5957 | | -1.55 | | | .12 | 8023 | | 2417 | | -1.23 | .22 | | | |
| 6. It is difficult to guide the call through the establishment of the video contact | 4885.5 | 5554.5 | | .11 | | | .91 | 8164 | | 2276 | | -.51 | .61 | | | |
| 7. Callers accept the use of video | 4787.5 | 5652.5 | | -.30 | | | .76 | 8149 | | 2291 | | -.62 | .54 | | | |
| 8. When I use video and get ‘eyes on the scene’, I am more able to assess the patient and help the caller | 5026.5 | 5413.5 | | .83 | | | .40 | 8231.5 | | 2208.5 | | -.20 | .84 | | | |
| 9. Video provides value in my visitation | 4917.5 | 5522.5 | | .28 | | | .78 | 7972.5 | | 2467.5 | | -1.68 | .09 | | | |
| 10. When I use video, I find it harder to forget the incident again | 4379 | 6061 | | -1.97 | | | .05 | 8668.5 | | 1771.5 | | 2.04 | .04 | | | |
| 11. I get more psychologically/emotionally affected then I assess an emergency call with video (compared to calls without video) | 4361 | 6079 | | -2.04 | | | .04 | 8657.5 | | 1782.5 | | 1.98 | .05 | | | |
| 12. I deselect video on emergency calls due to the risk of being emotionally affected | 4882 | 5558 | | .12 | | | .90 | 8454 | | 1968 | | 1.15 | .25 | | | |
| 13. It is unethical to use video during emergency calls | 4536 | 5904 | | -1.46 | | | .14 | 8559 | | 1881 | | 1.64 | .10 | | | |
| 14. When I use video, I experience that the situation is less dramatic than the caller describes | 4849.5 | 5590.5 | | -.03 | | | .97 | 7788.5 | | 2651.5 | | -2.46 | .01 | | | |
| 15. My conception of the situation is worse without video (where I only get the situation described by the caller) | 5060 | 5380 | | .83 | | | .41 | 8162.5 | | 2277.5 | | -.52 | .61 | | | |
| 16. When I use video, I have a better chance of de-escalating conflict situations | 4824 | 5616 | | -.14 | | | .89 | 8371.5 | | 2068.5 | | .54 | .59 | | | |
| 17. Video can support my guidance and dispatch in emergency calls where the issue/problem is unclear. | 4834.5 | 5605.5 | | -.11 | | | .91 | 8122 | | 2318 | | -.87 | .39 | | | |
| 18. It is a priority for the management in my organization that we use video | 4630.5 | 5809.5 | | -1.00 | | | .32 | 8371.5 | | 2068.5 | | .58 | .56 | | | |
| 19. I feel competent to use video | 4758.5 | 5681.5 | | -.51 | | | .61 | 8326.5 | | 2113.5 | | .39 | .70 | | | |
| 20. I feel motivated to use video | 4821.5 | 5618.5 | | -.16 | | | .87 | 8248 | | 2192 | | -.09 | .92 | | | |
| 21. My use of video has decreased over time | 5587.5 | 5952.5 | | -1.51 | | | .13 | 8291 | | 2149 | | .13 | .90 | | | |
| 22. When I use video, the duration of the emergency call is too long | 4221 | 6219 | | -2.60 | | | .01 | 8393 | | 2047 | | .64 | .52 | | | |
| 23. Video during emergency calls is suitable for all situations | 5069 | 5371 | | .86 | | | .39 | 7890.5 | | 2549.5 | | -1.88 | .06 | | | |

*This question was only possible to answer if the EMS dispatcher was employed before the implementation of video (*N* = 134).

**Supplementary Table 2**

*Mean Score on Questionnaire Items divided by the five EMDCs in Denmark on a scale from 1 'highly disagree' to 7 'highly agree' (N = 144).*

|  | **Northern Region** | **Central Denmark Region** | **Southern Region** | **Zealand Region** | **Capitol Region** |
| --- | --- | --- | --- | --- | --- |
| 1. After the introduction, I was able to use video during emergency calls | 6.80 | 6.92 | 6.80 | 6.46 | 6.38 |
| 2. It is easy to use video during emergency calls | 6.44 | 6.50 | 6.20 | 6.04 | 5.91 |
| 3. The technology for video during emergency calls works well | 4.56 | 4.71 | 4.60 | 4.75 | 5.14 |
| 4. It has been challenging to change my usual workflow when implementing video | 3.55 | 3.67 | 3.15 | 3.74 | 3.21 |
| 5. It is difficult to tell the caller that I would like to use video during the call | 2.32 | 2.13 | 2.47 | 2.58 | 2.88 |
| 6. It is difficult to guide the call through the establishment of the video contact | 2.60 | 3.54 | 3.67 | 3.38 | 3.48 |
| 7. Callers accept the use of video | 6.40 | 6.38 | 5.80 | 5.92 | 6.27 |
| 8. When I use video and get ‘eyes on the scene’, I am more able to assess the patient and help the caller | 6.24 | 6.33 | 6.67 | 6.50 | 6.64 |
| 9. Video provides value in my visitation | 6.12 | 6.38 | 6.53 | 6.38 | 6.68 |
| 10. When I use video, I find it harder to forget the incident again | 3.64 | 2.71 | 3.07 | 2.29 | 3.27 |
| 11. I get more psychologically/emotionally affected then I assess an emergency call with video (compared to calls without video) | 3.64 | 2.88 | 2.93 | 2.33 | 3.16 |
| 12. I deselect video on emergency calls due to the risk of being emotionally affected | 2.08 | 1.29 | 2.07 | 1.54 | 1.43 |
| 13. It is unethical to use video during emergency calls | 1.76 | 1.54 | 1.67 | 1.79 | 1.96 |
| 14. When I use video, I experience that the situation is less dramatic than the caller describes | 5.04 | 5.58 | 5.40 | 5.50 | 5.84 |
| 15. My conception of the situation is worse without video (where I only get the situation described by the caller) | 4.48 | 4.42 | 4.13 | 4.25 | 4.43 |
| 16. When I use video, I have a better chance of de-escalating conflict situations | 5.20 | 5.71 | 4.73 | 5.75 | 5.50 |
| 17. Video can support my guidance and dispatch in emergency calls where the issue/problem is unclear. | 6.52 | 6.50 | 6.33 | 6.54 | 6.75 |
| 18. It is a priority for the management in my organization that we use video | 6.32 | 6.83 | 5.20 | 5.42 | 6.43 |
| 19. I feel competent to use video | 6.72 | 6.67 | 6.40 | 6.38 | 6.71 |
| 20. I feel motivated to use video | 3.28 | 6.38 | 6.00 | 6.25 | 6.48 |
| 21. My use of video has decreased over time | 2.80 | 3.33 | 2.93 | 3.58 | 2.73 |
| 22. When I use video, the duration of the emergency call is too long | 4.92 | 3.38 | 3.60 | 4.21 | 3.96 |
| 23. Video during emergency calls is suitable for all situations | 2.56 | 3.13 | 3.40 | 3.08 | 3.00 |

*This question was only possible to answer if the EMS dispatcher was employed before the implementation of video (*N* = 134).

**Supplementary Table 3**

*Bivariate Correlations between Individual Items of the Questionnaire (N = 144).*

|  | **1** | **2** | **3** | **4** | **5** | **6** | **7** | **8** | **9** | **10** | **11** | **12** | **13** | **14** | **15** | **16** | **17** | **18** | **19** | **20** | **21** | **22** |
| --- | --- | --- | --- | --- | --- | --- | --- | --- | --- | --- | --- | --- | --- | --- | --- | --- | --- | --- | --- | --- | --- | --- |
| 1. After the introduction, I was able to use video during emergency calls |  |  |  |  |  |  |  |  |  |  |  |  |  |  |  |  |  |  |  |  |  |  |
| 2. It is easy to use video during emergency calls | .43 |  |  |  |  |  |  |  |  |  |  |  |  |  |  |  |  |  |  |  |  |  |
| 3. The technology for video during emergency calls works well | .21 | .43 |  |  |  |  |  |  |  |  |  |  |  |  |  |  |  |  |  |  |  |  |
| 4. It has been challenging to change my usual workflow when implementing video | -.16 | -.04 | .18 |  |  |  |  |  |  |  |  |  |  |  |  |  |  |  |  |  |  |  |
| 5. It is difficult to tell the caller that I would like to use video during the call | -.04 | -.16 | -.02 | .18 |  |  |  |  |  |  |  |  |  |  |  |  |  |  |  |  |  |  |
| 6. It is difficult to guide the call through the establishment of the video contact | -.09 | -.28 | -.25 | .11 | .55 |  |  |  |  |  |  |  |  |  |  |  |  |  |  |  |  |  |
| 7. Callers accept the use of video | .19 | .22 | .14 | -.15 | -.24 | -.18 |  |  |  |  |  |  |  |  |  |  |  |  |  |  |  |  |
| 8. When I use video and get ‘eyes on the scene’, I am more able to assess the patient and help the caller | .09 | .25 | .15 | -.19 | .05 | -.06 | .13 |  |  |  |  |  |  |  |  |  |  |  |  |  |  |  |
| 9. Video provides value in my visitation | .18 | .26 | .18 | -.24 | .04 | -.11 | .16 | .84 |  |  |  |  |  |  |  |  |  |  |  |  |  |  |
| 10. When I use video, I find it harder to forget the incident again | -.10 | -.02 | .00 | .15 | .09 | .08 | -.08 | -.02 | -.05 |  |  |  |  |  |  |  |  |  |  |  |  |  |
| 11. I get more psychologically/emotionally affected then I assess an emergency call with video (compared to calls without video) | -.13 | -.04 | -.06 | .20 | .10 | .10 | -.08 | -.08 | -.09 | .89 |  |  |  |  |  |  |  |  |  |  |  |  |
| 12. I deselect video on emergency calls due to the risk of being emotionally affected | -.05 | -.05 | .00 | .22 | .11 | .02 | -.04 | -.19 | -.24 | .36 | .38 |  |  |  |  |  |  |  |  |  |  |  |
| 13. It is unethical to use video during emergency calls | -.11 | -.26 | -.03 | .11 | .16 | .18 | -.12 | -.37 | -.35 | .27 | .22 | .37 |  |  |  |  |  |  |  |  |  |  |
| 14. When I use video, I experience that the situation is less dramatic than the caller describes | -.07 | -.06 | -.03 | -.25 | -.06 | -.00 | .01 | .13 | .12 | -.06 | -.06 | -.13 | -.11 |  |  |  |  |  |  |  |  |  |
| 15. My conception of the situation is worse without video (where I only get the situation described by the caller) | -.00 | .09 | .03 | -.17 | -.10 | -.10 | .16 | .20 | .11 | .01 | -.05 | -.07 | .02 | .30 |  |  |  |  |  |  |  |  |

| 16. When I use video, I have a better chance of de-escalating conflict situations | .06 | .04 | .04 | -.17 | -.13 | -.06 | .16 | .29 | .28 | .01 | -.02 | -.15 | -.08 | .27 | .14 |  |  |  |  |  |  |  |
| --- | --- | --- | --- | --- | --- | --- | --- | --- | --- | --- | --- | --- | --- | --- | --- | --- | --- | --- | --- | --- | --- | --- |
| 17. Video can support my guidance and dispatch in emergency calls where the issue/problem is unclear. | -.01 | .17 | .03 | -.26 | -.10 | -.11 | .21 | .38 | .35 | -.07 | -.13 | -.34 | -.29 | .06 | .02 | .30 |  |  |  |  |  |  |
| 18. It is a priority for the management in my organization that we use video | .05 | .12 | .03 | -.14 | -.06 | -.13 | .20 | .06 | .09 | .14 | .03 | -.17 | -.06 | .13 | .16 | .17 | .33 |  |  |  |  |  |
| 19. I feel competent to use video | .28 | .45 | .17 | -.27 | -.10 | -.16 | .34 | .34 | .41 | -.01 | -.02 | -.36 | -.36 | .03 | .19 | .11 | .42 | .32 |  |  |  |  |
| 20. I feel motivated to use video | .21 | .46 | .21 | -.26 | -.05 | -.15 | .30 | .66 | .66 | .07 | .03 | -.14 | -.27 | .04 | .22 | .26 | .49 | .31 | .66 |  |  |  |
| 21. My use of video has decreased over time | -.08 | -.25 | -.10 | .40 | .15 | .15 | -.15 | -.26 | -.22 | -.03 | .02 | .10 | .11 | -.01 | -.25 | -.17 | -.20 | -.20 | -.28 | -.31 |  |  |
| 22. When I use video, the duration of the emergency call is too long | -.17 | -.36 | -.30 | .17 | .20 | .25 | -.18 | -.32 | .35 | .10 | .11 | .16 | .21- | -.07 | -.10 | -.13 | -.22 | -.01 | -.21 | -.35 | .32 |  |
| 23. Video during emergency calls is suitable for all situations | -.01 | .13 | 23 | -.08 | -.23 | -.13 | .01 | .17 | .20 | -.09 | -.15 | -.08 | .18 | .11 | .25 | .16 | .15 | .11 | .13 | .20 | -.13 | -.20 |

*Note*. * *p <* .05; ** *p <* .01; *** *p <*.001. For question 4, *N* = 134.
