# Supplementary material for: Medical dispatchers’ experience with live video during emergency calls: a national questionnaire study
Source: BMC Health Serv Res. 2024 Nov 20;24:1442. doi: 10.1186/s12913-024-11939-4 (PMC11577824; doi:10.1186/s12913-024-11939-4)
Supplement: Supplementary file 2 — Supplementary material 2. [file 12913_2024_11939_MOESM2_ESM.docx]

Dear EMS Dispatcher,

This questionnaire is part of a national survey regarding the use of video in 1-1-2 calls. Your response to the questionnaire is anonymous. In addition to quality assurance, this national survey will also be used in a research project. Therefore, we kindly ask you to provide consent for participation on the next page. If you do not wish to consent to participation, you may close the browser window to withdraw from the survey.

**Consent form and information obligation for your participation in the Research Project:** **National survey on the use of video during medical emergency calls**

In relation to your participation in the national survey, we must inform you, in accordance with the General Data Protection Regulation, about how your personal data will be processed. This is a research project developed in collaboration between all five regional Emergency Medical Dispatch Centers (EMDCs) in Denmark, with the Central Denmark Region (CDR) being the data controller.

**Purpose and Legal Basis**: CDR processes both ordinary and sensitive personal data about you when necessary for the research project, which is carried out in the public interest and is of significant societal importance.

**Purpose:** The personal data is used and processed in the research project, which aims to investigate the use of video in 1-1-2 calls through a national questionnaire survey sent to all five EMDCs in Denmark.

**Legal Basis:** Section 10 of the Danish Data Protection Act.

**Source of Personal Data:** Your own responses in the questionnaire.

**Use of Personal Data:** The collected personal data is used for the research purposes described in the research project and may be disclosed to or further processed by others, e.g., for other research activities in accordance with data protection regulations and/or provisions in other legislation.

**Types of Data Processed:** All categories of personal data are processed in the research project, provided that the processing is objective, necessary, and relevant. For this research project, the following data is processed: Gender, age, educational background, seniority in the AMK-Vagtcentral, and responses to the questions.

**Transfer to Recipients in Third Countries, Including International Organizations:** If it is necessary for the research purpose to transfer personal data outside the EU and EEA, it will be done according to the rules of the General Data Protection Regulation, which aims to maintain the same level of security as in the EU and EEA. However, as a rule, we do not transfer your personal data to recipients outside the EU and EEA.

**Data Retention Period:** At this time, we cannot specify how long your personal data will be processed. Your personal data will be processed in a personally identifiable form as long as it is necessary for the research purposes and in accordance with the rules on retention after responsible research practices. When your personal data is no longer necessary for the processing, it will be anonymized, transferred to the Danish National Archives, or deleted.

**Your Rights:** When Region Midtjylland processes your personal data for scientific and statistical purposes, there are a number of limitations to the rights under the General Data Protection Regulation. This means, among other things, that you do not have the right to access, rectify, restrict, object to, or delete personal data about you that is included in such studies. The limitation of your rights follows from Section 22, Subsection 5 of the Danish Data Protection Act and Article 17, Subsection 3, Letter d of the General Data Protection Regulation.

**Complaints:** You have the right to lodge a complaint with the Danish Data Protection Agency if you believe Region Midtjylland is not processing your data correctly according to data protection legislation. You should always first contact Region Midtjylland if you are dissatisfied with how we process your personal data. You can read more about the possibility of lodging a complaint on the Danish Data Protection Agency's website: <https://www.datatilsynet.dk/borger/klage/saadan-klager-du>

**Data Controller Authorities/Contact Information:** Region Midtjylland, Skottenborg 26, 8800 Viborg In Region Midtjylland, the research project is led by Natascha Hougaard Bohnstedt-Pedersen, who can be contacted at Tel: +45 4296 5214 or Email: natboh@rm.dk. The research project is a collaboration between all five AMK-Vagtcentraler in Denmark, and a project manager from your own region can also be contacted.

**Contact Information for the Data Protection Officer (DPO):** Email: dpo@rm.dk

**Consent to Participation:** By responding to the questionnaire, you indicate that you agree to participate in the national survey on the use of video in 1-1-2 calls.

I consent to participate in the research project: National Survey on the Use of Video in 1-1-2 Calls.

**Basic information details**

| Which EMDC do you work at? | - Central Denmark Region - Northern Denmark Region - Southern Denmark Region - Region Zealand - The Capital Region |
| --- | --- |
| Your professional background | - Nurse - Paramedic - Ambulance Technician - Other |
|  | If other, please specify:  ________________________ |
| Seniority in the EMDC | - < 1 year - 1 year - 2 years - 3 years - 4 years - 5 years - 6 years - 7 years - 8 years - 9 years - ≥ 10 years |
| Your age | - 18 - 19 - 20 - 21 - 22 - 23 - 24 - 25 - 26 - 27 - 28 - 29 - 30 - 31 - 32 - 33 - 34 - 35 - 36 - 37 - 38 - 39 - 40 - 41 - 42 - 43 - 44 - 45 - 46 - 47 - 48 - 49 - 50 - 51 - 52 - 53 - 54 - 55 - 56 - 57 - 58 - 59 - 60 - 61 - 62 - 63 - 64 - 65 - 66 - 67 - 68 - 69 - 70 - 71 - 72 - 73 - 74 - 75 - 76 - 77 - 78 - 79 - 80 |
| Gender | - Female - Male - Other |

**Questions regarding the use of video during medical emergency calls**

| I have been able to use a video solution during emergency calls for | - 0 – 6 months - 7 – 12 months - 1 – 2 years - 2 – 3 years - > 3 years - I was employed after the introduction of a video solution |
| --- | --- |
| I assess, that I ______ use video during emergency calls | - Always - Often - Sometimes - Rarely - Never |

**Please answer the following questions by selecting the option that best reflects your experience.**

The questions are based on a comparison between emergency calls with video and emergency calls without video.

|  | Strongly agree |  |  | Neutral |  |  | Strongly disagree |
| --- | --- | --- | --- | --- | --- | --- | --- |
| **After the introduction, I was able to use video during emergency calls** |  |  |  |  |  |  |  |
| **It is easy to use video during emergency calls** |  |  |  |  |  |  |  |
| **The technology for video during emergency call works well** |  |  |  |  |  |  |  |
| **Is has been challenging to change my usual workflow when implementing video** |  |  |  |  |  |  |  |
| **It is difficult to tell the caller that I would like to use video during the call** |  |  |  |  |  |  |  |
| **It is difficult to guide the caller through the establishment of the video contact** |  |  |  |  |  |  |  |
| **Callers accept the use of video** |  |  |  |  |  |  |  |

**Experiences, impacts and opportunities with video**

|  | Strongly agree |  |  | Neutral |  |  | Strongly disagree |
| --- | --- | --- | --- | --- | --- | --- | --- |
| **When I use video and get ’eyes on the scene’, I am more able to assess the patient and help the caller** |  |  |  |  |  |  |  |
| **Video provides value in my visitation** |  |  |  |  |  |  |  |
| **When I use video, I find it harder to forget the incident agian** |  |  |  |  |  |  |  |
| **I get more psychologically/emotionally affected when I assess an emergency call with video (compared to calls without video)** |  |  |  |  |  |  |  |
| **I deselect video on emergency calls due to the risk of being emotionally affected** |  |  |  |  |  |  |  |
| **It is unethical to use video during emergency calls** |  |  |  |  |  |  |  |
| **When I use video, I experience that the situation is less dramatic than the caller describes** |  |  |  |  |  |  |  |
| **My conception of the situation is worse without video (where I only receive the situation described by the caller)** |  |  |  |  |  |  |  |
| **When I use video, I have a better chance of de-escalating conflict situations** |  |  |  |  |  |  |  |
| **Video can support my guidance and dispatch in emergency calls where the issue/problem is unclear** |  |  |  |  |  |  |  |

**The use of video**

|  | Strongly agree |  |  | Neutral |  |  | Strongly disagree |
| --- | --- | --- | --- | --- | --- | --- | --- |
| **It is a priority for the management in my organization that we use video** |  |  |  |  |  |  |  |
| **I feel competent to use video** |  |  |  |  |  |  |  |
| **I feel motivated to use video** |  |  |  |  |  |  |  |
| **My use of video has decreased over time** |  |  |  |  |  |  |  |
| **When I use video, the duration of the emergency call is too long** |  |  |  |  |  |  |  |
| **Video during emergency calls is suitable for all situations** |  |  |  |  |  |  |  |

**Greatest barrier for using video during emergency calls**

| **What is the main reason for you not to use video during medical emergency calls? (select only one)** | - It is difficult to introduce video to the caller during the emergency call - Video does not give me more value in my assessment and dispatch - Video is not suitable in all situations occurring in emergency calls - I experience psychological overload when using video - I find it harder to forget the incident again when I use video - The introduction to use video was deficient - Missing management prioritization in using video - I do not have the competences to use video - I do not have the motivation to use video - I do not maintain my use of video - Using video lengthens the duration of the emergency call - I experience difficulties with the technology - It is unethical to use video during emergency calls - I do not need to use video to have a good feeling of the situation - I do not wish to answer |
| --- | --- |

**If you have any additional comments or
feedback, you can write them here:**

____________________________________________________
